# *Artemisia scoparia* and Metabolic Health: Untapped Potential of an Ancient Remedy for Modern Use

**DOI:** 10.3389/fendo.2021.727061

**Published:** 2022-02-08

**Authors:** Anik Boudreau, Allison J. Richard, Innocence Harvey, Jacqueline M. Stephens

**Affiliations:** ^1^ Adipocyte Biology Laboratory, Pennington Biomedical Research Center, Baton Rouge, LA, United States; ^2^ Department of Biological Sciences, Louisiana State University, Baton Rouge, LA, United States

**Keywords:** *Artemisia scoparia*, diabetes, inflammation, adipocyte, botanical, ethnophamacology

## Abstract

Botanicals have a long history of medicinal use for a multitude of ailments, and many modern pharmaceuticals were originally isolated from plants or derived from phytochemicals. Among these, artemisinin, first isolated from *Artemisia annua*, is the foundation for standard anti-malarial therapies. Plants of the genus *Artemisia* are among the most common herbal remedies across Asia and Central Europe. The species *Artemisia scoparia* (SCOPA) is widely used in traditional folk medicine for various liver diseases and inflammatory conditions, as well as for infections, fever, pain, cancer, and diabetes. Modern *in vivo* and *in vitro* studies have now investigated SCOPA’s effects on these pathologies and its ability to mitigate hepatotoxicity, oxidative stress, obesity, diabetes, and other disease states. This review focuses on the effects of SCOPA that are particularly relevant to metabolic health. Indeed, in recent years, an ethanolic extract of SCOPA has been shown to enhance differentiation of cultured adipocytes and to share some properties of thiazolidinediones (TZDs), a class of insulin-sensitizing agonists of the adipogenic transcription factor PPARγ. In a mouse model of diet-induced obesity, SCOPA diet supplementation lowered fasting insulin and glucose levels, while inducing metabolically favorable changes in adipose tissue and liver. These observations are consistent with many lines of evidence from various tissues and cell types known to contribute to metabolic homeostasis, including immune cells, hepatocytes, and pancreatic beta-cells. Compounds belonging to several classes of phytochemicals have been implicated in these effects, and we provide an overview of these bioactives. The ongoing global epidemics of obesity and metabolic disease clearly require novel therapeutic approaches. While the mechanisms involved in SCOPA’s effects on metabolic, anti-inflammatory, and oxidative stress pathways are not fully characterized, current data support further investigation of this plant and its bioactives as potential therapeutic agents in obesity-related metabolic dysfunction and many other conditions.

## Introduction

Rising obesity rates around the globe are driving an epidemic of metabolic syndrome (MS) and type 2 diabetes (T2DM), and novel therapeutic interventions are needed. Because the pathogenesis of obesity-related metabolic dysfunction is multifactorial and complex, diverse strategies have been employed to hinder its development and manifestations, namely stimulating insulin production in pancreatic beta-cells, inhibiting hepatic glucose output, reducing glucose reabsorption in the kidney, and enhancing peripheral glucose uptake and insulin sensitivity (1). The molecular mechanisms driving these effects include inhibition of ATP-sensitive potassium channels in pancreatic beta-cells to stimulate insulin release, activation of the glucagon-like peptide-1 (GLP-1) receptor or inhibition of dipeptidyl peptidase-4 activity to enhance incretin signaling and lower circulating glucose levels, and activation of the peroxisome proliferator-activated receptor-gamma (PPARγ) in adipocytes to improve insulin sensitivity in peripheral tissues (2–5). Type 1 diabetes (T1DM), which represents only about five percent of diabetes mellitus cases, results from the progressive destruction of pancreatic beta-cells and consequent inability to produce insulin. Therefore, interventions to improve insulin sensitivity are ineffective for T1DM, and insulin replacement is currently the only glucose-lowering pharmacological treatment available (6). In addition to strategies for controlling glycemia, treatment of both T1DM and T2DM also includes management of diabetic complications such as kidney disease, cardiovascular disease, and retinopathy.

The first-line medication for T2DM, metformin, is a synthetic derivative of the phytochemical galegine, first isolated from *Galega officinalis.* This plant, also known as French lilac or goat’s rue, was used medicinally in medieval Europe for many ailments, including symptoms that are now attributed to T2DM (7). Metformin reduces hepatic glucose production *via* mechanisms that have not been fully elucidated. While it is known that metformin activates adenosine monophosphate (AMP)-activated protein kinase (AMPK) in the liver, there is evidence that several AMPK-independent mechanisms are likely to be involved in its metabolic impacts (4, 8). These include inhibition of mitochondrial respiration and of the gluconeogenic pathway (8–12). In addition, metformin’s glucose-lowering activity may be partially mediated through effects on the gut (13–15). Therefore, although metformin has been used clinically for over half a century, there is still significant debate around its precise mechanisms of action.

Across the world, plants have been used medicinally for centuries, and many pharmaceuticals are derived from natural products. Even now, factors such as availability, cost, or cultural practices drive the continued use of botanical products as supplements or alternatives to pharmaceuticals. Although rigorous and thorough investigation is often lacking, many plants are currently being screened or studied both *in vitro* and *in vivo* to assess their bioactivities and efficacy. One such plant, *Artemisia scoparia* (SCOPA), has a long history of medicinal use in much of Asia and Central Europe to treat liver diseases, inflammatory conditions, and diabetes, among other ailments. The genus *Artemisia* comprises hundreds of species, some of which are among the most widely used medicinal plants across the world (1, 2). Perhaps the best known product of the genus is the anti-malarial drug artemisinin, whose isolation from *Artemisia annua* was awarded the Nobel Prize for Physiology or Medicine in 2015 (3). Other medicinal species include *A. capillaris, A. absinthum, A. argyi, A. capillaris* and *A. dracunculus*, but there are many more (4–6). Modern studies have now established that extracts from SCOPA exert a wide range of effects in many cell types and animal models. In addition, many individual bioactive compounds responsible for these effects have been identified. This review describes the traditional folk medicine uses of SCOPA and examines what is currently known about its effects in various animal models and cell types, with a focus on findings relevant to metabolic health. We also discuss individual compounds in SCOPA and their wide range of effects, including the potential to attenuate metabolic dysfunction, particularly in the context of diet-induced obesity. Although SCOPA has not been studied in T1DM, some of its reported actions suggest that it may mitigate diabetic complications in addition to improving glycemic control. Such effects could be beneficial in T1DM as well as T2DM.

Even in the absence of a fully elucidated mechanism of action, identifying additional agents, like metformin, from natural products with therapeutic potential against metabolic dysfunction is of great value in fighting the growing epidemics of obesity, MS, and T2DM. Thus, an overarching goal of this review is to compile and evaluate anecdotal and mechanistic studies of *A. scoparia*’s ability to modulate metabolic function. We also aim to demonstrate the potential of bioactives from *A. scoparia* for modern clinical and/or complementary use to support metabolic health, while highlighting the significant need for additional studies to evaluate mechanism(s) of action on a molecular level.

## Ethnopharmacology and Traditional Medicinal Uses of *A. scoparia*

**Figure 1:**
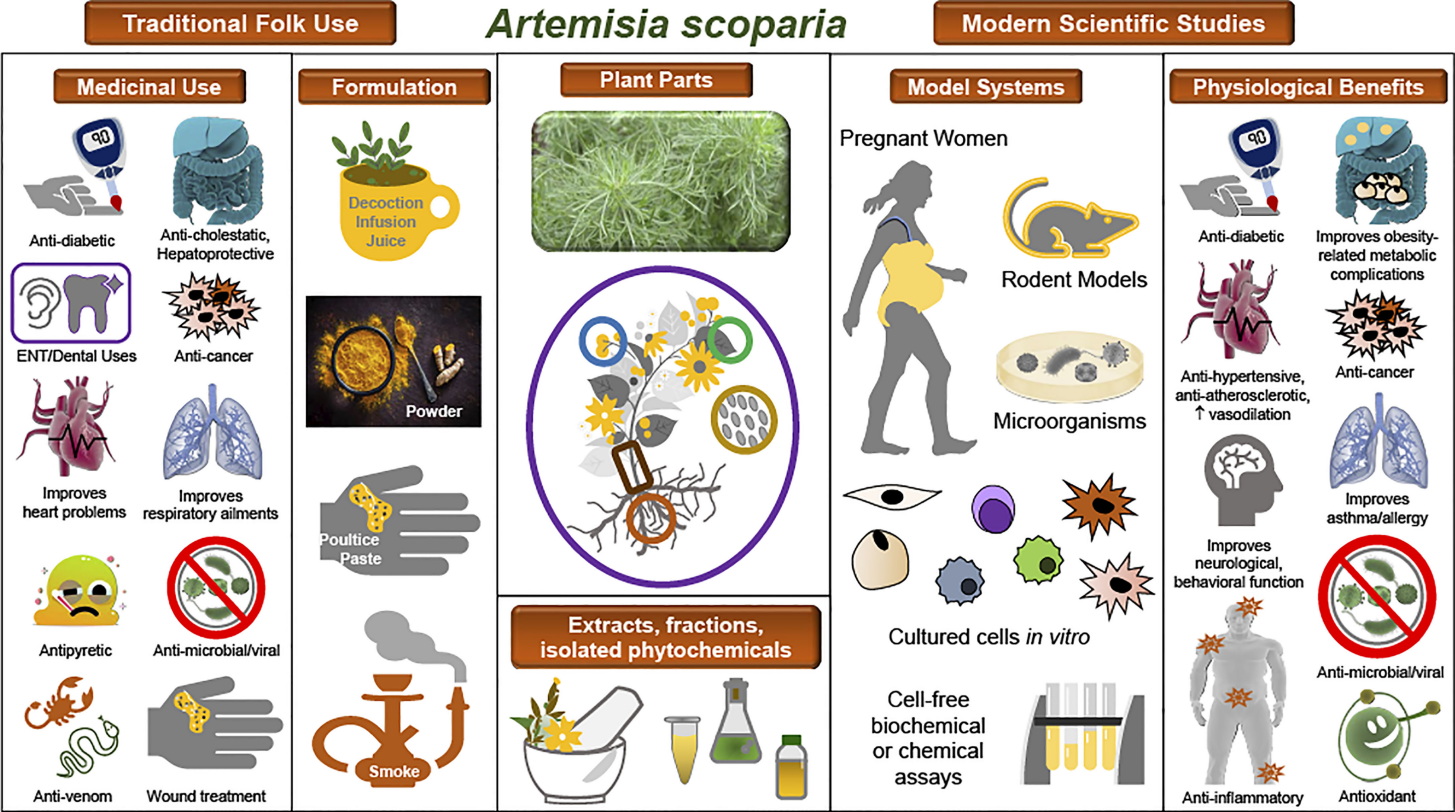
Traditional uses and observed biological effects of *A. scoparia* and its constituents. Illustration of the ethnopharmacology of *A. scoparia*, experimental models used in the study of its bioactivity, and its principal observed effects in pathophysiological conditions. Center: A photograph of *A. scoparia* is shown along with a diagram of the plant parts used in traditional medicine and in modern studies. For use or study of SCOPA, extracts, fractions, or isolated bioactive compounds have been obtained from its flowers, leaves, stems, roots, and seeds, as well as the whole plant. Left: Traditional folk medicine indications and formulations as documented in ethnobotanical studies. Right: Modern scientific studies have included numerous model systems, such as humans, rodents, and microorganisms; *in vitro* studies in differentiating and mature adipocytes, immune cells, and cancer cells; and cell-free assays of antioxidant or enzymatic activities. Data from scientific studies support some of the ethnopharmacology claims and reveal additional pathophysiologies that may benefit from use of SCOPA extracts or its isolated bioactive constituents.

SCOPA is one of the most widely used medicinal plants across many parts of Asia, and modern ethnobotany studies have documented its many indications in Afghanistan, Pakistan, Saudi Arabia, Iran, and China for conditions such as liver, gallbladder, and digestive disorders; various infectious and inflammatory diseases; ear pain; cardiovascular conditions; and diabetes and hyperglycemia (7–33). One example of the ethnopharmacological documentation of SCOPA is a study conducted in the Upper Neelum Valley of Pakistan, in which data collected from interviews were analyzed and individual plants or medicinal indications were assigned quantitative ethnobotanical indices. SCOPA was determined to have a high use value in this population (21). Reported indications and formulations for SCOPA in traditional folk medicine are shown in **Table 1** and **Figure 1**. It is notable that many of these are common to distinct populations in diverse regions.

**Table 1:** Documented indications and geographic locations for traditional uses of *A. scoparia*.

Medicinal Use	Region	Plant Part	Formulation	ROA^b^	Ref(s)
**Diabetes/ Hyperglycemia**	Pakistan/Afghanistan border	Root	Decoction	Oral	(10)
	Lower Kurram, Pakistan	Root			(28)
	Zhejiang Province, China	Root	Infusion	Oral	(23)
	Uttarakhand, India	Leaves	Powder	Oral	(34)
	Shigar Valley, Pakistan	Leaves	Infusion	Oral	(32)
**Cancer**	Pakistan/Afghanistan border	Root	Decoction	Oral	(10)
	China				(35)^a^
**Hepatitis, jaundice, liver or gallbladder disease**	Neelum Valley, Pakistan	Leaves	Infusion	Oral	(21)
	China				(35)^a^
	China	Aerial parts	Decoction	Oral	(36)
	Pakistan/Afghanistan border	Root	Decoction	Oral	(10)
	Onaizah Province, Saudi Arabia	Whole plant	Decoction	Oral	(37)
	Zhejiang Province, China	Flowers	Decoction	Oral	(23)
**Digestion**	Neelum Valley, Pakistan	Leaves	Infusion	Oral	(21)
	Hormozgan Province, Iran	Leaves	Decoction	Oral	(25)
	Ilam Province, Iran	Flowers		Internal	(16)
	China				(38)
	Pakistan				(39)
	Samahni Valley, Pakistan	Leaves, roots	Juice, Decoction	Oral	(27)
	Onaizah Province, Saudi Arabia	Whole Plant	Decoction	Oral	(37)
	Zhejiang Province, China	Root	Infusion	Oral	(23)
	Swat District, Pakistan	Young shoots			(19)
	Spiti Valley, Western Himalaya, India				(20)
	Uttarakhand, India	Leaves	Powder	Oral	(34)
	Gujranwala District, Pakistan	Whole plant	Powder, roasted	Oral	(13)
**ENT/Dental**	Pakistan			Topical	(26, 39)
	Ilam Province Iran (Kurd tribe)	Flowers		Internal	(16)
	Samahni Valley, Pakistan	Leaves, roots	Juice, decoction	Topical	(27)
	Onaizah Province, Saudi Arabia	Whole plant	Decoction	Ear drops	(37)
	Spiti Valley, Western Himalaya, India	Leaves, seeds	Poultice	Topical	(20)
**Depurative “blood purification”**	Lower Kurram, Pakistan	Roots	Decoction	Oral	(28)
	Pakistan		Infusion	Oral	(26, 39)
	Samahni Valley, Pakistan	Leaves, roots	Juice, decoction	Oral	(27)
	Uttarakhand, India	Leaves	Powder	Oral	(34)
**Fever, microbial or parasitic infections, snake or scorpion venom**	Pakistan				(39)
	China				(38)
	Pakistan	Whole plant			(40)
	Zhejiang Province, China	Leaves	Decoction	Oral	(23)
	Swat District, Pakistan	Whole plant	Decoction	Oral	(17)
	Swat District, Pakistan	Young shoots			(19)
	Uttarakhand, India	Leaves	Powder, roasted	Oral	(34)
	Gujranwala District, Pakistan	Leaves		Topical	(13)
	Gujranwala District, Pakistan	Flowers, shoots	Decoction	Oral	(13)
**Burns/wounds/skin/hair**	Pakistan	Twigs	Smoke	External	(26, 39)
	Ilam Province, Iran	Flowers		Internal	(16)
	Spiti Valley, India		Smoke	External	(20)
	Uttarakhand, India	Leaves	Paste	Topical	(34)
	Gujranwala District, Pakistan	Leaves	Extract+oil, boiled	Topical	(13)
**Cardiovascular**	Pakistan	Whole plant			(40)
	China				(38)
	Onaizah Province, Saudi Arabia	Whole plant	Decoction	Oral	(37)
	Zhejiang Province, China	Roots	Infusion	Oral	(23)
**Respiratory**	Pakistan				(39)
	Pakistan	Whole plant			(40)
	Zhejiang Province, China	Leaves	Decoction	Oral	(23)
	Uttarakhand, India	Leaves	Powder, roasted	Oral	(34)

a Yinchen – refers to A. scoparia or A. capillaris.

b ROA, Route of Administration.

SCOPA is very prominent in Traditional Chinese Medicine (TCM), particularly for its hepatoprotective and choleretic effects. Its uses are extensively described in the canon of TCM literature (41–44) and cited in the current Chinese Pharmacopoeia (35). “Artemisia scopariae herba” (ASH), or “Yinchen”, refers to the dried aerial parts of SCOPA or of its close relative, *Artemisia capillaris*, with the two plants being used interchangeably for its preparation. A decoction of Yinchen is the principal constituent of many TCM formulae, in which ASH is combined with other herbal products. Importantly, a distinction is made between ASH from small Spring seedlings (Mian Yin Chen) and that from flowering plants in late Summer (Yen Chin Hao), each preferable for treating distinct sets of ailments (41, 45). Chemical analyses of ASH have confirmed that its constituents vary greatly depending on the time of harvest (36, 46). While TCM preparations are derived from the aerial parts of SCOPA, the roots of the plant are used in Pakistan (27) and the use of its flowers has been reported in Iran (16).

## Effects of *A. scoparia* on Determinants of Metabolic Health

### Metabolically Favorable Effects of *A. scoparia* in Experimental Models of Obesity and Diabetes

As mentioned above, the use of SCOPA in folk medicine for diabetes and hyperglycemia has been amply documented. A 2016 study found that administration of SCOPA during the second trimester of pregnancy improved insulin sensitivity, fasting plasma glucose levels, and circulating adiponectin levels in patients with gestational diabetes (47). With the exception of that study, SCOPA has not been evaluated in humans for effects on measures of metabolic health such as insulin sensitivity, glycemic control, or cardiovascular risk factors. Likewise, few animal studies have been conducted to examine SCOPA*’*s metabolically relevant effects. However, in a mouse model of diet-induced obesity (DIO) and insulin resistance, SCOPA administration by gavage or supplementation of diet was found to improve insulin sensitivity as measured by homeostatic model assessment for insulin resistance (HOMA-IR) or insulin tolerance test (ITT) (48–50). Circulating levels of triglycerides, free fatty acids (FFAs), glycerol, and insulin (fasting) were reduced, while adiponectin levels were increased (49–51). In liver, SCOPA supplementation reduced hepatic triglyceride and cholesterol content and enhanced insulin-induced phosphorylation of the signaling proteins insulin receptor substrate (IRS-1), insulin receptor subunit beta (IRβ), protein kinase B (AKT1), and RAC-beta serine/threonine protein kinase (AKT2) (50). Moreover, adenosine monophosphate (AMP)-activated protein kinase (AMPK) activity was enhanced and expression levels of genes involved in *de novo* lipogenesis were reduced by SCOPA in liver, consistent with the observed improvements in hepatic lipid accumulation (50). SCOPA also had pronounced effects in adipose tissue (AT), where it was shown to robustly enhance insulin-induced phosphorylation of AKT protein in epididymal, but not retroperitoneal or inguinal, white adipose tissue (WAT) depots (49). Levels of monocyte chemoattractant protein 1 (MCP-1), an inflammatory cytokine known to be highly expressed in obesity and insulin resistance, were also significantly reduced in SCOPA-treated animals (48).

### Pro- and Anti-Adipogenic, and Anti-Lipolytic Effects of *A. scoparia* in Adipocytes

As described above, an ethanolic extract of SCOPA has metabolically favorable effects in a mouse model of DIO, including improvements in adipose tissue function. This same extract has been shown to enhance adipogenesis of 3T3-L1 cells, a widely used model to study adipocyte differentiation, as measured by both lipid accumulation and adipogenic gene expression (48). A recent study has revealed that SCOPA can promote adipogenesis in the absence of 3-isobutyl-1-methylxanthine (MIX), a key component in the classic adipocyte differentiation cocktail, and SCOPA significantly induces the expression of several PPARγ target genes, also regulated by MIX, that enhance lipid accumulation during adipogenesis. These data suggest that SCOPA’s adipogenic effects are partially mediated by increased PPARγ activity (52). Another research group investigating individual compounds isolated from a different SCOPA extract reported that 4 of the 19 compounds tested significantly inhibited lipid accumulation in 3T3-L1 cells during differentiation, while other compounds enhanced adipocyte development (53). In this study, no total or parent extracts of SCOPA were tested. A third laboratory observed inhibition of lipid accumulation in 3T3-L1 cells with their crude SCOPA extract as well (54). These apparent discrepancies illustrate three great challenges that accompany the study of botanical extracts: Individual compounds have complex interactions and often fail to mirror effects observed with the parent extracts; plants from different geographic regions, grown in different conditions or harvested at different times of the year may have very different chemical compositions; and variable extraction methods across studies make it impossible to confidently compare results.

Given that obesity drives insulin resistance and diabetes, interventions to reduce fat mass have been pursued as a means to counter obesity-associated metabolic disease. Lowering adiposity through increased energy expenditure or reductions in food intake can indeed have favorable metabolic effects. However, adipogenesis is typically impaired, not enhanced, in obese and insulin resistant states, and limiting adipose tissue expansion in conditions of positive energy balance by inhibiting adipocyte development is generally considered detrimental, as it promotes dyslipidemia and ectopic lipid accumulation (55). This point is underscored by the fact that drugs such as the thiazolidinediones (TZDs), which stimulate adipogenesis *via* PPARγ activation, are potent insulin sensitizers (56). Since TZDs have fallen out of use in recent years due to significant side effects, efforts are ongoing to identify natural product partial agonists of PPARγ to combat metabolic syndrome (57–60). Researchers investigating SCOPA in adipocytes have employed these alternate strategies (inhibition or promotion of adipogenesis) and have therefore focused on different bioactivities in SCOPA. It should be noted that unlike the pro-adipogenic extract described above, SCOPA extracts that were found to inhibit adipogenesis have not been evaluated *in vivo* for effects on insulin sensitivity, lipid metabolism, or glycemic control.

Obesity and insulin resistance result in abnormally high rates of lipolysis in the fed state, driven by the impaired action of insulin to inhibit lipolysis, as well as by the chronic inflammation characteristic of obese states (61). As mentioned previously, SCOPA supplementation in the food of high-fat diet-fed mice lowered circulating FFAs and glycerol, consistent with reduced lipolysis rates in adipose tissue (62). In cultured adipocytes, inflammation-associated lipolysis was inhibited in the presence of this same SCOPA extract, indicating that SCOPA has cell-autonomous antilipolytic activity in adipocytes. Interestingly, lipolysis induced by adrenergic stimulation or unstimulated basal lipolysis were not altered by SCOPA (62). A different SCOPA extract has been shown to modestly increase lipolysis in adipocytes under basal conditions but was not tested in inflammatory conditions (63). Given that unstimulated lipolysis rates are very low, this observation may not be relevant in the context of obesity, where inflammatory cytokines drive high lipolysis rates.

### Hepatoprotective Effects of *A. scoparia*

Liver and gallbladder conditions including jaundice and cholestasis are among the illnesses most commonly treated with Yinchen (*A. scoparia* or *A. capillaris*). Research aimed at characterizing these hepatoprotective and choleretic properties has focused mostly on TCM formulations containing Yinchen in combination with other herbs, or on individual compounds isolated from Yinchen, rather than on the *Artemisia* extracts. However, two studies by Gilani et al. have demonstrated that SCOPA extract could attenuate liver injury induced by acetaminophen in mice (64) or by carbon tetrachloride in rats (65). Hepatic glucose output and lipid metabolism are major contributors to the regulation of circulating glucose and lipid levels, and thus liver function is key in preserving metabolic homeostasis. Likewise, metabolic dysregulation in obesity can lead to ectopic lipid accumulation in liver and non-alcoholic fatty liver disease (NAFLD). It is therefore plausible that beneficial effects of SCOPA in liver could preserve glycemic control and maintain appropriate circulating lipid levels in conditions of hepatic stress or, conversely, protect the liver from the deleterious effects of obesity and insulin resistance. This is supported by the mouse DIO study described above, in which SCOPA improved insulin sensitivity and reduced hepatic lipid accumulation (50).

### Anti-Inflammatory and Antioxidant Effects of *A. scoparia*

Obesity and T2DM are considered inflammatory states. Infiltration of macrophages and altered resident immune cell populations in adipose tissue promote inflammation and insulin resistance (66, 67). Many conditions treated by SCOPA in TCM or folk medicine have an inflammatory component [(41) and **Table 1**], and SCOPA has been shown to have anti-inflammatory properties in a wide range of conditions, including inhibition of heat-induced protein denaturation *in vitro* (68) and reducing inflammatory cytokine production, cell infiltration, and edema in carrageenan-induced acute inflammation in rats and mice (69, 70). Similarly, topical application of SCOPA diminished clinical symptoms, cell infiltration, inflammatory cytokine levels, caspase-1 activity in lesions, and circulating levels of histamine in a mouse model of atopic dermatitis (71). Reductions in markers of adipose tissue inflammation in DIO mice have also been observed with SCOPA supplementation (48, 49). In addition, there are abundant data showing anti-inflammatory actions of SCOPA in cultured cell lines relevant to metabolic function. In lipopolysaccharide (LPS)-stimulated RAW 246.7 murine macrophages, an ethanolic SCOPA extract, previously found to attenuate lipolysis and markers of adipose tissue inflammation, also inhibited the expression of several inflammatory genes (72). In this same cell line, a different ethanolic extract reduced nitric oxide (NO) production in cells treated with LPS and interferon gamma (IFNγ) (53), while a methanolic extract from a third source failed to inhibit NO release from LPS-treated RAW 264.7 cells (63). Although the reason for this discrepancy cannot be ascertained, the three studies investigated extracts prepared from different plant material originating from diverse geographic locations, using various extraction methods and solvents, and tested at different doses; one or more of these factors could explain these seemingly conflicting results. Similar studies were conducted in isolated bone marrow-derived macrophages (BMDM) from mice, in which NO, inducible nitric oxide synthase (iNOS), and inflammatory cytokine levels were all reduced by SCOPA treatment in stimulated cells (69). Comparable effects of SCOPA have been observed in the THP-1 human monocyte cell line, undifferentiated 3T3-L1 murine preadipocytes, and in the HMC-1 human mast cell line (69, 73). Moreover, reduced pro-inflammatory NF-κB promoter activation in IL-1β-treated pancreatic beta-cells, which are also vulnerable to obesity-related inflammation, has been observed in response to SCOPA (72). Taken together, these data from multiple models and treatment conditions clearly indicate that SCOPA is a potent anti-inflammatory agent and that it can antagonize inflammation in conditions consistent with metabolic dysregulation.

Oxidative stress plays an important role in insulin resistance, the progression to diabetes, and diabetic complications. Indeed, hyperglycemia induces the production of reactive oxygen and nitrogen species, and the resulting oxidation of lipids, proteins, and DNA mediates diabetic complications such as neuropathy, nephropathy, retinopathy, and vascular damage. Although oxidative stress occurs in response to hyperglycemia, it can also drive metabolic dysfunction, as it hinders insulin signaling and glucose uptake in cultured adipocytes, myocytes, and vascular smooth muscle cells (74–80). Mechanisms involved in these effects have been attributed to mitochondrial dysfunction, inhibition of insulin signaling proteins, and negative modulation of the expression and translocation of the glucose transporter GLUT-4 [reviewed in (80)]. Furthermore, reactive oxygen species (ROS) have a range of deleterious effects on pancreatic beta-cell function, including increased apoptosis, reduced beta-cell neogenesis, mitochondrial dysfunction, and impaired insulin secretion (78, 81). Finally, oxidative stress can contribute to insulin resistance by activating inflammatory pathways (80). Essential oils and extracts of SCOPA have been reported to have antioxidant and free radical-scavenging properties (82–85), which could be consistent with improvements in metabolic function. Studies and reviews of SCOPA’s anti-inflammatory and antioxidant effects are shown in **Table 2**.

**Table 2:** *In vitro* and *in vivo* studies of *A. scoparia*.

Extract	Metabolic Complications	Cardiovascular/dyslipidemia	Hepatic dysfunction	Cancer	Inflammation/oxidative stress	Neurological/Behavioral	Anti-microbial	Renal
**Ethanolic extract**	(48–50, 62, 72, 86)	(62)			(72)			
**Methanolic extract**	(63)			(63, 87)	(83, 84)			(84)
**Aqueous Extract or fraction**		(88)			(68, 69)	(89)		
**Total flavonoid**					(68, 90)			
**Essential oil**					(82, 91)		(92)	
**DCM****^a^**** extract**							(83)	
**Commercial extract, n.s.****^b^**	(47)							
**Crude extract**	(54)		(65)		(68)			
**Whole extract, n.s.****^b^** **or butanol fraction**					(71)			

a DCM, dichloromethane.

b n.s., Not specified.

### Cardiovascular Effects of *A. scoparia*

Metabolic syndrome is a cluster of risk factors for cardiovascular disease and diabetes. Obesity, insulin resistance, and diabetes promote hypertension, hyperlipidemia, and vascular damage, thereby increasing risks of coronary artery disease, stroke, and peripheral vascular disease. SCOPA has been used as an anti-hypertensive, a vasodilator, and an anti-hypercholesterolemic agent in traditional medicine (**Table 1**), and data from modern studies have been consistent with these historical uses. As is the case for SCOPA’s hepatoprotective properties, investigations have focused on SCOPA-containing TCM preparations or on single compounds isolated from SCOPA, but Cho et al. have shown that diet supplementation with an aqueous SCOPA extract lowered blood pressure and produced other favorable effects in spontaneously hypertensive rats (88). Beneficial effects included reductions in angiotensin converting enzyme (ACE) activity, angiotensin II (AngII) levels, and lipid peroxidation in serum. Given these observations, it is plausible that SCOPA’s effects on the cardiovascular system could mitigate some of the complications of metabolic syndrome or diabetes. Studies showing cardiovascular effects of SCOPA appear in **Table 2**.

## Effects of Bioactive Compounds Found in *A. scoparia*

### Coumarins

SCOPA is rich in plant coumarins (93). The three related coumarins scoparone (6,7 - dimethoxycoumarin), scopoletin (7-hydroxy-5-methoxycoumarin), and esculetin (6,7 - dihydroxycoumarin) are found in many *Artemisia* species and are considered major components of SCOPA (36, 41). Many coumarins have potent anti-inflammatory or antioxidant effects that account for a wide range of bioactivities (94). In addition, natural and synthetic coumarins are under investigation as promising treatments for many conditions, cancer in particular (95–102). The therapeutic potential of these coumarins is supported by molecular docking analyses (94, 103–112) and structure-activity relationship (SAR) studies (111, 113–117).

#### Scoparone

Scoparone, a prominent compound in TCM preparations, has long been known to have hypotensive and vasodilatory properties (118–125) and has been found to have several additional favorable cardiovascular effects, including inhibition of ACE activity *in vitro* (126) and reduction in AngII-induced myocardial changes in rodents, cultured myocytes, and cardiac fibroblasts (127, 128). Furthermore, there is evidence that scoparone can mitigate cardiac ischemia/reperfusion injury in *in vitro* and *in vivo* settings (129). Scoparone also has anti-atherogenic effects including the inhibition of vascular smooth muscle cell proliferation and migration (130, 131), inhibition of platelet aggregation (132), and the attenuation of atherosclerotic plaque formation and dyslipidemia in hyperlipidemic diabetic rabbits (133, 134). One study also found that scoparone decreased peroxisome proliferator-activated receptor gamma (PPARγ) activity, expression of PPARγ target genes, and lipid accumulation in differentiating 3T3-L1 adipocytes (135). In cultured rat mesangial cells, high glucose-induced production of extracellular matrix proteins was reduced with scoparone treatment (136), a finding with potential implications for renal diabetic complications.

As is the case for SCOPA extracts and traditional preparations, hepatoprotective effects have been reported for scoparone in NASH (137, 138) and in conditions of hepatotoxicity or liver injury induced by carbon tetrachloride or alcohol (139–141). Consistent with folk uses of SCOPA in the treatment of jaundice and cholestasis, scoparone also promotes bilirubin clearance through activation of the constitutive androstane receptor (CAR) (142). Interestingly, CAR activation has been shown to improve insulin sensitivity, glucose metabolism, and hepatic lipid accumulation in leptin-deficient *ob/ob* mice (143), and to prevent obesity, insulin resistance, and hepatic steatosis in HFD-fed mice (144). CAR agonism has been proposed as a therapeutic target for obesity, insulin resistance, and diabetes (142–146). In a recent study, a panel of natural and synthetic coumarin derivatives was screened for the ability to activate CAR, and scoparone was found to improve glucose tolerance in leptin receptor-null *db/db* mice (147).

Anti-inflammatory and antioxidant properties of scoparone have been demonstrated in a wide range of conditions, and some of the pathways impacted by these effects have been described. Studies in the murine RAW 264.7 macrophage cell line demonstrated that scoparone could attenuate the LPS- or IFNγ-induced production of inflammatory cytokines, as well as iNOS and cyclooxygenase 2 (COX2) protein levels and corresponding NO and prostaglandin E2 (PGE2) release (148). Similar results were obtained in a human monocyte cell line, in which scoparone attenuated phorbol-12-myristate-13-acetate (PMA)-induced inflammatory cytokine production by inhibiting nuclear factor kappa-light-chain-enhancer of activated B cells (NF-κB) activation (149).

In a mouse model of acute lung injury, pulmonary edema, histological changes, and LPS-mediated inflammatory cytokine production were improved by scoparone *in vivo*, while *in vitro* experiments in alveolar macrophages revealed that the compound’s anti-inflammatory effects were mediated through the toll-like receptor 4 (TLR4)/NFκB pathway (150). Anti-inflammatory effects of scoparone in a rat model of colitis have also been reported (151), while in BV2 microglial cells, scoparone attenuated LPS-induced neuroinflammatory responses by blocking interferon regulatory factor 3 (IRF3) and extracellular signal-regulated kinase (ERK) activation (126). In a mouse model of acute seizures, scoparone preserved blood-brain barrier integrity, prevented inflammation and apoptosis, and inactivated the phosphoinositide 3-kinase (PI3K)/AKT signaling pathway *in vivo.* This same study determined that scoparone could also inhibit astrocyte activation elicited by LPS (152).

In addition to interfering with inflammatory pathways, scoparone has been shown to provide protection from oxidative stress, as demonstrated in 2,2-diphenyl-1-picrylhydrazyl (DPPH) and lipid peroxidation assays *in vitro* (151, 153), and to inhibit production of ROS and preserve antioxidant enzyme activity in response to several types of oxidative stressors (154, 155). Antioxidant activity has also been implicated in scoparone’s ability to protect against kidney damage elicited by the chemotherapeutic agent cisplatin (156), to reduce markers of pancreatic fibrosis in cultured pancreatic stellate cells (157), and to prevent osteoclast differentiation and bone resorption *in vitro* (158).

Effects of scoparone have been observed in many other systems and models, including immunosuppressive functions associated with autoimmunity, allergies, and graft rejection (159, 160); neurite outgrowth and dopamine synthesis and release in the PC12 neuronal cell line (161–163); proliferation and migration of cancer cells (105, 164); bactericidal, antifungal, and antiparasitic properties (165–167); promotion of melanogenesis; and activation of the cystic fibrosis transmembrane conductance regulator (CFTR) (168, 169). **Table 3** presents a summary of the wide range of bioactivities attributed to scoparone.

**Table 3:** *In vitro* and *in vivo* effects of bioactive compounds found in *A. scoparia*.

Compound	Metabolic Complications	Cardiovascular/dyslipidemia	Hepatic dysfunction	Cancer	Inflammation/oxidative stress	Neurological/Behavioral	Anti-microbial	Renal	Reproduction	Asthma/Allergy	Other
**Coumarins**											
Scoparone	(132–136, 147)	(119, 122–134)	(137, 138, 140, 142, 154, 170–174)	(105, 164)	(149–151, 153–155, 157, 175, 176)	(126, 152, 161–163)	(165–167)	(136, 156)	(177)	(159, 160, 175)	(178)^a^ (158, 179);^b^ (169);^c^
Scopoletin	(108, 116, 180–190)	(126, 132, 191–199)	(154, 200–202)	(97, 203–213)	(114, 154, 214–245)	(106, 109, 110, 227, 246–264)	(165, 265–278)		(279)	(175, 251, 280–288)	(289, 290)^d^ (291–293);^b^ (294, 295);^e^ (296–298);^a^
Esculetin	(116, 180, 299–312)	(303, 313–317)	(139, 171, 318–320)	(96, 321–349)	(226, 307, 318, 350–366)	(111, 367–372)	(165, 314, 373–375)	(107, 339, 376–378)	(379)	(282, 354, 380–383)	(384–386)^b^ (387, 388);^f^ (389);^g^
**Flavonoids**											
Flavonols^h^	(57, 390–397)	(395, 398–402)	(395, 403, 404)	(395, 405, 406)	(395, 407)	(393, 395)	(395, 408)	(395)		(395)	(395, 409)
Rutin	(394, 397, 410–416)	(402, 417)	(412, 415, 418–421)	(422, 423)	(419, 420, 424, 425)			(418, 420)	(418, 420)	(417)	(421)^d^ (412, 425);^e^
Flavanones^i^	(426–429)	(427, 430, 431)	(432)	(427, 433)	(427, 428, 434)		(435, 436)				(429)^b^ (437);^c^
**Chromones**											
Capillarisins	(116)		(438, 439)	(440–443)	(444–449)	(448)			(450)	(451)	(452)^g^
**Phenolic Acids**											
Chlorogenic Acids^j^	(453–455)	(455–458)	(459)	(460, 461)	(71, 73, 222, 462–464)	(465–467)	(468–470)				(471)^d^
Prenylated coumaric acids^k^	(472–477)			(478–485)	(464, 472, 486–489)		(490, 491)			(492)	(493)^d^ (488);^e^ (494);^l^

a Melanogenesis.

b Osteoprotection.

c Respiratory.

d Aging and healthspan.

e Gastrointestinal.

f Ophthalmic.

g Cartilage or muscle.

h Quercetin and Isorhamnetin.

i Naringenin and Blumeatin.

j Caffeic acid, Dicaffeoylquinic acids, Chlorogenic acid.

k Artepillins, Capillartemisins, Drupanin, Scopa-coumaricins.

l Hypoxia/ischemia.

#### Scopoletin

Scopoletin is a naturally fluorescent compound found in many plants (191, 214, 215, 246). It is a substrate for peroxidases, which convert scopoletin to non-fluorescent compounds, and has thus been widely used for many decades, in combination with horseradish peroxidase, as the basis for high-sensitivity hydrogen peroxide detection assays (495–498). Several studies have demonstrated scopoletin’s antioxidant capabilities *in vitro*, using common cell-free methods such as the DPPH, Trolox equivalent antioxidant capacity (TEAC), ferric reducing ability of plasma (FRAP), or beta-carotene/bleaching assays, while superoxide, hydrogen peroxide, nitric oxide, and peroxynitrite are among the reactive species shown to be effectively scavenged by scopoletin (114, 180, 216–223, 247). Hepatoprotective antioxidant effects of scopoletin have also been observed in cultured HepG2 cells and primary hepatocytes (154, 200), however one study failed to detect significant antioxidant activity for scopoletin (499).

Given the important role of oxidative stress in the etiology of numerous disorders, it is not surprising that some of scopoletin’s favorable effects in disease states are attributed to its antioxidant properties. For example, scopoletin has been reported to have antioxidant effects in hyperthyroid-induced hyperglycemia in rats (224), as well as in oxidant-induced hemolysis of rat erythrocytes (225). The brain is particularly vulnerable to oxidative stress, which is known to be central to many neurodegenerative conditions (500). A study performed in mouse brain homogenates revealed that scopoletin strongly inhibited the oxidative protein modifications induced by copper (226), which can contribute to the pathologies associated with atherosclerosis, Alzheimer’s disease (AD), and Wilson’s disease (501–503). A recent study examined several aspects of oxidative stress involved in the pathogenesis of Parkinson’s disease (PD) and showed that scopoletin attenuated depletion of cellular reduced glutathione or ATP, inhibited ROS generation, and prevented cell death in oxidative conditions *in vitro* (227). These findings were extended to a *Drosophila* mutant model of PD, where scopoletin treatment reduced accumulation of mitochondrial ROS and promoted recovery from degenerative phenotypes (227). Several other studies have shown that scopoletin can prevent oxidative injury in models relevant to diseases such as PD and AD. These include prevention of oxidative injury and induction of antioxidant gene expression in HT-22 and SHSY-5Y cells (248, 249), as well as inhibition of monoamine oxidase activity (106, 250).

One important contributor to oxidative stress in many cell types is the xanthine oxidase (XO)/xanthine dehydrogenase (XDH) system (201, 504, 505). XO activity in the liver produces uric acid, which is released into circulation and excreted by the kidney. Hyperuricemia, due to excessive uric acid production in the liver or impaired clearance in the kidney, causes accumulation of uric acid crystals in joints (gout) and in the kidney. Scopoletin shows inhibitory activity in enzymatic assays of XO *in vitro* (506). Additionally, scopoletin administered either by intraperitoneal injection or by oral gavage of scopoletin-loaded micelles was shown to correct hyperuricemia in mice through two separate mechanisms, namely inhibiting hepatic XO activity and enhancing uric acid excretion by the kidney (201, 202). Notably, XO activity is associated with obesity-related metabolic dysfunction, and XO inhibitors typically prescribed for gout or hyperuricemia are proving to be effective in mitigating cardiovascular and renal complications of diabetes (504, 507, 508).

In addition to antioxidant effects, scopoletin has potent anti-inflammatory activity. Numerous studies have demonstrated the ability of scopoletin to diminish the production of proinflammatory mediators such as cytokines and eicosanoids in many cell types, including macrophages, mast cells, fibroblasts, and platelets (228–237). *In vivo* effects of scopoletin in rodent ear or paw edema models and in models of inflammatory conditions such as arthritis, gastro-esophageal disease, gastric ulcers, gout, pleurisy, pancreatitis, as well as nociceptive or analgesic properties, have also been described (215, 237–242, 291, 509, 510). Mechanisms involved in scopoletin’s anti-inflammatory effects include negative regulation of inflammatory signaling pathways and inhibition of lipoxygenase and cyclooxygenase enzyme activities (214, 239, 243, 247, 280, 281). Scopoletin’s anti-inflammatory properties have also been implicated in its effects on various pathologies, particularly in the realm of immunity, as it has been shown to regulate complement pathway activation, mast cell degranulation, as well as several aspects of innate, humoral, and adaptive immune function, suggesting potential roles in allergies, asthma, and autoimmune diseases such as multiple sclerosis and rheumatoid arthritis (175, 251, 282–288, 511).

Scopoletin has been studied in several models of neurological dysfunction and has an array of favorable effects. Some of these are at least partly attributable to its antioxidant or anti-inflammatory properties, like reducing inflammation-induced anxiety in mice or attenuating neural deficits, brain edema, and inflammatory cytokine production in intracerebral hemorrhage in rats (252, 253). In addition, several groups have demonstrated scopoletin’s ability to inhibit acetyl- and butyryl-cholinesterases *in vitro* (110, 247, 254–257, 512) and *in vivo* (258). Antidepressant, anti-psychotic, and anti-amnesic effects were also observed in behavioral studies in mice (246, 258–261). A study investigating anticonvulsant effects of *Benkara malabarica* (Linn.) root extract found that scopoletin inhibited GABA transaminase activity (262). Consistent with this finding, molecular docking analysis has demonstrated affinity of scopoletin for both GABA transaminase and for the GABA-A receptor (252). The anticonvulsant drug vigabatrin is an irreversible inhibitor of GABA-T, and there is convincing evidence that it is effective in attenuating anxiety symptoms (513). Formation of amyloid beta peptide 42 (Aβ42) and α-synuclein fibrils, processes central to the pathogenesis of AD and PD respectively, have both been shown to be inhibited by scopoletin (109, 110). These actions are likely complementary to the antioxidant mechanisms described above (227) in combatting neurodegenerative diseases. Finally, scopoletin improves neuronal plasticity, as measured in *ex vivo* electrophysiological assays, and exerts neuroprotective activity in a rat spinal cord injury model (263, 264).

In conditions related to metabolic health, scopoletin acts favorably on many pathways *in vivo*, as well as in various cell types and experimental conditions. In animal models of diet-induced obesity, diabetes, or alcohol-induced metabolic dysfunctions, scopoletin restored insulin sensitivity, reversed disruptions in circulating lipids, glucose, insulin, and inflammatory cytokines, while also attenuating lipid accumulation and fibrosis in liver, restoring adiponectin levels in white adipose tissue, and reducing oxidative stress in the pancreas (181–187). Consistent with these *in vivo* observations, scopoletin has been shown to mitigate insulin resistance and improve metabolic functions in cultured hepatocytes, adipocytes, and pancreatic beta-cells (184, 185, 188, 514). *In vitro* assays have revealed that enzymes involved in glucose homeostasis (protein tyrosine phosphatase 1b, α-glucosidase and α-amylase) or in diabetic complications (aldone reductase) are inhibited by scopoletin (108, 116, 180, 184, 187, 189, 190). While effects described above are likely to improve cardiovascular health in the context of metabolic syndrome, scopoletin also acts directly on the heart and vasculature. Scopoletin has antihypertensive actions (126, 191–193), anti-atherosclerotic capabilities (132, 194–196), and vascular spasmolytic/vasodilatory effects (191, 193, 197–199).

Scopoletin is a compound of interest in cancer research, as it has been shown to have apoptotic, cytotoxic, anti-proliferative, anti-angiogenic, and anti-metastatic activities in various cancer cell lines (97, 203–212). Efforts are ongoing to develop synthetic derivatives of scopoletin and to characterize and improve its bioavailability and pharmacokinetic properties (96). Finally, a handful of other bioactivities have been reported for scopoletin, among them the promotion of melanogenesis (296–298) and osteoprotective (292, 293), antitussive (515), gastrokinetic (294), and antimicrobial properties (165, 265–278, 516), as well as anti-aging effects in skin and lung fibroblasts (289, 290).

It is notable that many of the same signaling pathways are modulated by scopoletin across the wide variety of experimental models and conditions in which it has been investigated. Scopoletin can activate the AMPK pathway (181, 182, 186, 263, 514) and the PI3K/AKT pathway (184, 186, 188, 514), and inhibit the inflammatory TLR4/myeloid differentiation factor 88 (MyD88) and NF-κB pathways (94, 181, 183, 233, 237, 241, 251, 252, 517, 518). There is also substantial evidence that scopoletin can inhibit the MAPK pathway in a variety of cell types (199, 235, 237, 241, 252, 290, 297).

#### Esculetin

Like scoparone and scopoletin, esculetin’s well-documented antioxidant and anti-inflammatory properties are central to its beneficial effects in biological systems (226, 350–355, 384, 519). A broad range of inflammatory or oxidative conditions have been shown to be impacted by esculetin. These include lung injury and fibrosis (356, 357, 520); fibromyalgia (521); neuronal oxidative stress (522); psoriasis (523); arthritis (358); nociception (359); colitis (360); allergy, immunity, and asthma (282, 354, 380–383); and sepsis (361). The effects of esculetin on numerous cancer cell types have been extensively studied (94, 321–342, 519, 524, 525) and are the basis for efforts to develop novel therapeutics (98, 341). Neuroprotective and behavioral actions of esculetin have also been described (111, 299, 367–371, 526–528). Selected publications highlighting these effects of esculetin are featured in **Table 3**.

### Flavonoids and Chromones

Flavonoids are highly abundant compounds in all plants; they are extensively studied for various bioactivities and have a wide range of effects in many biological systems. Numerous flavonoids have been identified in SCOPA, however their relative abundance varies widely among plants and extracts from different sources (36, 41). Since there exists abundant literature regarding the bioactivities of flavonoids, we will present only a subset of the most commonly reported, most abundant, or most thoroughly investigated flavonoids in SCOPA. As a representative example, cirsmaritin has been shown to have anti-proliferative, anti-metastatic, and anti-carcinogenic effects in cancer cell lines (529–534), diabetes- and metabolism-related effects (535–537), as well as anti-inflammatory, antioxidant, and antimicrobial properties (538–543). Studies have also demonstrated that cirsimaritin can modulate neurological, immune, and digestive functions (544–548), and attenuate renal injury (549, 550). Additional activities of selected flavonoids are shown in **Table 3**.

The chromone capillarisin is known as a major constituent of *Artemisia capillaris.* It is also relatively abundant in SCOPA but is not known to be a bioactive constituent of other plants. There are multiple reports of anti-inflammatory and antioxidant effects of capillarisin (444–448, 551), which has also been shown to inhibit tumor cell invasion, inhibit signaling transducer and activator of transcription 3 (STAT3) activation, slow cell growth, and promote apoptosis in various cancer cell lines (440, 441, 443, 552). Other reported effects of capillarisin include anti-asthmatic activity (451) and promotion of penile erection in a rabbit model (450). Studies of capillarisin and its derivatives that are found in SCOPA are summarized in **Table 3**.

### Phenolic Acids

Chlorogenic acid, caffeic acid, 3,5-dicaffeoylquinic acid, and 4,5-dicaffeoylquinic acid are related phenolic acids found in many plants or, in the case of caffeic acid, all plants. Chlorogenic acid and its derivatives are abundant in SCOPA and have been shown to mediate some of SCOPA’s effects (71, 73, 85). Unlike the ubiquitous chlorogenic acid derivatives, prenylated coumaric acids (PCAs) are not common in plants, and the most thoroughly characterized of these compounds, artepillin C, drupanin, and baccharin, have been primarily studied from Brazilian green propolis (476, 553–555). The most prominent bioactivities of chlorogenic acids and PCAs are presented in **Table 3**. Although not all studies of SCOPA’s chemical constituents have detected PCAs, capillartemisin B and drupanin have been identified in SCOPA (53, 556–558), and additional PCAs have been reported in *Artemisia capillaris* (559, 560). PCAs are not considered major constituents of SCOPA, however several of them have been isolated from a SCOPA extract with potent adipogenic activity (556, 557). Fractions of this extract that were most effective in promoting adipogenesis were rich in PCAs, and activity was confirmed for three PCAs isolated from these fractions, including two co-purified isomers of a novel PCA, termed “cis-scopa-trans-coumaricin” and “cis-scopa-cis-coumaricin”. Like the PCAs from SCOPA, PCAs from propolis can activate PPARγ, promote adipocyte differentiation, and mitigate obesity-associated metabolic dysfunction (472–474, 561–563). Given that these compounds have not been reported in SCOPA extracts found to inhibit adipogenesis, the presence or absence of PCAs is a plausible explanation for the seemingly divergent effects of SCOPA on adipocytes in studies using different extract preparations. Interestingly, a unique enzyme has recently been isolated from *A. capillaris* that catalyzes two successive prenylations of p-coumaric acid to yield artepillin C, with drupanin as a mono-prenylated intermediate (564). It appears likely that an equivalent enzyme may exist in SCOPA and in other plants that synthesize these compounds.

## Bridging the Gap: SCOPA as a Modern Intervention to Promote Metabolic Health

We have reviewed multiple lines of evidence for metabolic benefits of SCOPA. However, these data have emerged in a piecemeal fashion, and comprehensive studies to adequately assess SCOPA as a therapeutic or preventive intervention for metabolic dysfunction are lacking. One ethanolic SCOPA extract has been shown to have beneficial effects on adipose tissue function, hepatic lipid accumulation, and insulin sensitivity in a mouse model of DIO (48–50), and a different extract was found to attenuate gestational diabetes in a small human study (47). Ethnopharmacological data, as well as the broad range of bioactivities of SCOPA observed in various cell lines and disease models, also provide strong rationale to investigate SCOPA in humans with obesity or metabolic syndrome. The widespread medicinal use of SCOPA in many parts of the world suggests favorable safety and toxicity profiles, however these have not been formally studied, and such data will be needed in the assessment of SCOPA’s potential as a therapeutic intervention. For example, since SCOPA is known to affect hepatic function and to increase whole-body insulin sensitivity in mice, adverse effects in the liver or risk of hypoglycemia are potential concerns. Given the high variability in the chemical composition of SCOPA extracts, their rigorous characterization, using unbiased and standardized methods will also be crucial in interpreting results from different extracts and in guiding pharmacokinetic evaluation of potential therapeutic extracts. In our view, details of extract composition reported for SCOPA are generally insufficient, and greater efforts are required to characterize extracts that are being studied for the promotion of metabolic health. Repositories of serum and tissue samples from *in vivo* studies would also be helpful in laying the groundwork for pre-clinical or translational studies. Differences in biological effects and chemical composition among various SCOPA preparations could also serve as a resource for correlating constituent compounds with bioactivity.

SCOPA’s reported hepatoprotective and antioxidant properties, as well as its beneficial effects on cardiovascular parameters are consistent with favorable metabolic effects but have not been investigated in the context of human metabolic disease. In addition, SCOPA’s potential effects in many cell types and experimental models relevant to obesity or metabolic syndrome have not yet been interrogated. These include measures of insulin sensitivity or glucose uptake in skeletal muscle cells, glucose output from hepatocytes, and insulin secretion from pancreatic beta-cells. SCOPA’s ability to mitigate diabetic complications is also unknown, although its documented antioxidant and anti-inflammatory effects suggest that it may protect against the consequences of chronic hyperglycemia. All these aspects of SCOPA bioactivity merit more systematic assessment in conditions of metabolic dysfunction. Finally, mechanisms responsible for SCOPA’s pleiotropic actions remain only partially explored. As described in this review, SCOPA or its constituent compounds have been shown to regulate various signaling pathways or enzyme activities, but the molecular players, mechanistic details, and implications of these effects remain to be elucidated. Thus, a wide range of experimental observations offer promising evidence of SCOPA’s metabolic benefits, but critical pieces of data are needed to realize its full promise as a *bona fide* therapeutic.

## Conclusion

The historical and anthropological importance of botanicals in health and disease is unquestionable. Not only are plants used in folk medicine applications around the world, but they are also consumed as nutritional supplements and are the origin of many modern pharmaceuticals. Despite the successes of synthetic drug development, there is great value in investigating complex botanical extracts for several reasons. First, there is a need to characterize and evaluate botanicals in current use. A cross-sectional study conducted between 2002 and 2012 reported that 18% of adults in the US use dietary supplements (565). According to the American Botanical Council, sales of botanical supplements topped 8.8B$ in 2018 and were on the rise (abc.herbalgram.org). This market is largely unregulated, and rigorous studies addressing the safety, modes of action, and efficacy of such supplements are desirable. Second, synergistic interactions between phytochemical compounds are common, and individual constituents often fail to recapitulate activities of their parent botanical extracts (566, 567). Third, the thorough study of complex botanical extracts enables the identification of novel and unique lead compounds that may not otherwise emerge or that may be challenging to synthesize, as in the case of the PCAs reported in *Artemisia* species including SCOPA.

This review of SCOPA’s ethnopharmacology, bioactivities, and constituent compounds reveals a remarkable range of traditional uses and experimental data and provides a valuable example of both the potential and the difficulties of studying complex botanical extracts. Indeed, we have described promising *in vivo* and *in vitro* data supporting SCOPA’s use for many pathologies, in particular hepatic diseases and obesity-related metabolic dysfunction, as well as proven effects of individual compounds found in SCOPA. **Figure 1** illustrates the principal findings related to SCOPA. We have also presented seemingly contradictory data regarding the adipogenic effects of SCOPA, with studies showing both pro- (48, 49, 556, 557) and anti- (53, 54, 63) adipogenic activities. However, analysis of the pro-adipogenic extract and fractions revealed the presence of PCAs, known to promote adipogenesis, while these compounds were not reported in extracts that inhibited adipogenesis.

To be sure, investigation of botanical extracts is a challenging endeavor due to the enormous complexity of the mixtures, the considerable variability in extract composition from different plants of the same species (36, 46, 568, 569), the potential for unpredictable experimental artifacts (570), the imperfect methods for detecting constituent compounds, and the biased nature of investigations based on the research interests and priorities of different investigators. However, increasingly sophisticated preparatory, analytical, and computational methods are helping to overcome these difficulties. In order to enhance the reliability and translatability of natural products research, the National Center for Complementary and Integrative Health (NCCIH) has spearheaded the development of comprehensive good practices for pre-clinical investigation of natural products (567). We fully support these principles and encourage further study of the bioactivities of *Artemisia scoparia*, particularly in metabolism research, in accordance with these guidelines.

## Author Contributions

A.B. wrote the first draft of the manuscript and prepared the information for the tables. I.H wrote a small part of the review and edited the entire document. AR edited the document and prepared the final version of the Tables and Figure. JS worked with AB on original outline of the review and edited each draft. All authors contributed to the article and approved the submitted version.

## Funding

This publication was supported by the National Center for Complementary & Integrative Health and the Office of Dietary Supplements of the National Institutes of Health (NIH) under Award Number P50AT002776 which funds the Botanical Dietary Supplements Research Center of Pennington Biomedical Research Center and the Department of Plant Biology and Pathology in the School of Environmental and Biological Sciences (SEBS) of Rutgers University.

## Conflict of Interest

The authors declare that the research was conducted in the absence of any commercial or financial relationships that could be construed as a potential conflict of interest.

## Publisher’s Note

All claims expressed in this article are solely those of the authors and do not necessarily represent those of their affiliated organizations, or those of the publisher, the editors and the reviewers. Any product that may be evaluated in this article, or claim that may be made by its manufacturer, is not guaranteed or endorsed by the publisher.
